# Visible Exocytosis of the Non-Photic Signal Neuropeptide Y to the Suprachiasmatic Nucleus in Fasted Transgenic Mice Throughout Their Circadian Rhythms

**DOI:** 10.3390/bioengineering12020192

**Published:** 2025-02-17

**Authors:** Kazuo Nakazawa, Minako Matsuo, Kazuki Nakao, Shigenori Nonaka, Rika Numano

**Affiliations:** 1 Department of Applied Chemistry and Life Science, Toyohashi University of Technology, Toyohashi 441-8580, Japan; nakazawa.kazuo.ic@tut.jp; 2 Institute for Research on Next-generation Semiconductor and Sensing Science, Toyohashi University of Technology, Toyohashi 441-8580, Japan; 3 Laboratory of Animal Resources, Center for Disease Biology and Integrative Medicine, Graduate School of Medicine, The Osaka University, Suita 565-0871, Japan; 4 Spatiotemporal Regulations Group, Exploratory Research Center for Life and Living Systems (ExCELLS), Okazaki 444-8585, Japan; 5 Laboratory for Spatiotemporal Regulations, National Institute for Basic Biology, Okazaki 444-8585, Japan

**Keywords:** neuropeptide Y, feeding requirement, superchiasmatic nucleus, transgenic mice, NPY::Venus, secretory granules, circadian rhythms

## Abstract

Organisms maintain circadian rhythms corresponding to approximately 24 h in the absence of external environmental cues, and they synchronize the phases of their autonomous circadian clocks to light–dark cycles, feeding timing, and other factors. The suprachiasmatic nucleus (SCN) occupies the top position of the hierarchy in the mammalian circadian system and functions as a photic-dependent oscillator, while the food-entrainable circadian oscillator (FEO) entrains the clocks of the digestive peripheral tissues and behaviors according to feeding timing. In mammals, neuropeptide Y (NPY) from the intergeniculate leaflet (IGL) neurons projected onto the SCN plays an important role in entraining circadian rhythms to feeding conditions. However, the relationship between the FEO and SCN has been unclear under various feeding conditions. In this study, novel NPY::Venus transgenic (Tg) mice, which expressed the NPY fused to Venus fluorescent protein, were generated to investigate the secretion of NPY on the SCN from the IGL. NPY-containing secretory granules with Venus signals in the SCN slices of the Tg mice could be observed using confocal and super-resolution microscopy. We observed that the number of NPY secretory granules released on the SCNs increased during fasting, and these mice were valuable tools for further investigating the role of NPY secretion from the IGL to the SCN in mediating interactions between the FEO and the SCN.

## 1. Introduction

Living beings maintain their internal clocks in accordance with an approximately 24 h period, i.e., a circadian rhythm, and they synchronize the phases of their autonomous clocks with an environmental cycle even in the absence of daily external cues. The central pacemaker of the mammalian circadian rhythm is located in the SCN. Mammals reset the phases of their circadian rhythms in the SCN each morning, mainly according to environmental light signals. In individual cells, the molecular clock is driven by a feedback loop of the transcription and translation of clock genes such as *Period* (*Per1,2*) [1,2,3].

We created *Per1* promoter::luciferase (*Per1*::*luc*) transgenic (Tg) rats and mice for the real-time monitoring of *Per1* expression, and we observed the molecular rhythms in the different tissues because *Per1* is known as one of the key molecular regulators in the mammalian core clock system. Using these Tg rats, we showed that the SCN can reset the phase of a circadian clock using a photic signal according to light and dark environmental cues [4,5,6]. On the other hand, feeding schedules, such as a 4 h restricted feeding (RF) period during a subjective day, can synchronize the circadian clocks of the digestive system, liver, and other organs [7,8,9,10]. In addition, we maintained the *Per1*::*luc* Tg mice under ad libitum feeing (free feeding, FF) and RF conditions for 4 h (feeding them only at the Zeitgeber time (ZT) for 4–8 and 4–8 h after lights were turned on) under light conditions. RF could not entrain the phase shift of the *Per1*::*luc* bioluminescent rhythms in the SCN, whereas the phases of the digestive system and food-anticipatory activity (FAA) were reset by RF for 4 h (at ZT 4–8) during light conditions for at least 48 h [8].

Therefore, it was hypothesized that the food-entrainable circadian oscillator (FEO) transfers signals to the peripheral tissues of the digestive system and FAA independently of the SCN in response to feeding requirements. A potential signaling molecule of the FEO is neuropeptide Y (NPY), which can influence the circadian oscillator in the SCN [11,12,13,14,15,16,17,18,19]. When NPY is added to SCN slices, it induces a 2–5-h phase advance in the molecular clock of the SCN in vitro [11,12,13].

For NPY-deficient adult mice, entrainment of the phase of the circadian rhythm according to the timing of RF is repressed and the amount of food consumed during RF is reduced because NPY signaling plays a critical role in regulating feeding behavior rhythms under various feeding conditions [14]. The intergeniculate leaflet (IGL)–SCN circuit regulated by thalamic IGL neurons could entrain the phases of circadian rhythms to non-photic cues, such as RF. The SCN receives dense axonal projections from the IGL and NPY in orchestrated circadian systems [15,16,17,18]. In the absence of NPY, *Per1* and *Per2* in the SCN receive normal induction, but when more than normal NPY is projected, *Per1* and *Per2* induction is not inhibited. IGL neurons that synthesize NPY play an important role in preventing the FEO from connecting to the SCN network [15,19].

However, the spatiotemporal dynamics of NPY secretion around the SCN in response to feeding requirements remain unclear. In this study, we successfully developed a system for directly visualizing NPY secretion in Tg mouse brain slices. In future studies, researchers could perform real-time monitoring of food-entrained circadian clock function according to NPY secretion using NPY::Venus Tg mice [20,21,22]. The fluorescence signal of NPY::Venus increased in the SCN slices of the Tg mice during fasting. NPY may allow the FEO to work independently of the SCN and thereby modulate circadian rhythms in response to feeding requirements by repressing input photic signals [4,22,23,24,25].

## 2. Materials and Methods

### 2.1. Generation of the Tg Mice

**Figure 1:**
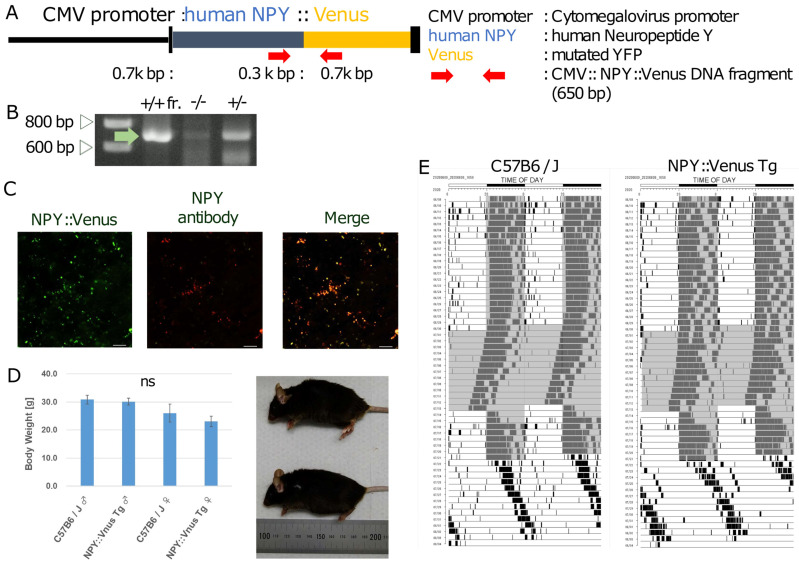
Generation of the NPY::Venus Tg mice. (**A**) Construction of the transgene with the CMV promoter linked to NPY::Venus that was used to generate the Tg mice. The red arrows indicate the primer pairs used to detect an NPY::Venus DNA fragment. (**B**) PCR screening of NPY::Venus using the genomic DNA of wild-type (−/−) and NPY::Venus Tg heterozygous (+/−) mice. The positive control (+/+) was plasmid DNA containing NPY::Venus. The 600 and 800 bp bands of the markers are shown in the lane labeled ‘M’. The green arrow indicates the 650 bp band amplified from the NPY::Venus DNA. (**C**) Fluorescent images of NPY::Venus (green) and the immunostaining of NPY with an anti-NPY primary antibody and secondary antibody (red) in an SCN slice of an NPY::Venus Tg mouse. The NPY and Venus signals were identified in the merged image (green and red). Scale bar: 10 µm. (**D**) Body weights and lengths between the NPY::Venus Tg and C57B6/J wild-type sibling mice. (**Left**) A bar graph showing the body weights of the male and female C57B6/J mice and the male and female NPY::Venus Tg mice. (*n* = 5). (**Right**) Body lengths between an NPY::Venus Tg male mouse (top) and a C57B6/J male mouse (bottom). (**E**) A double-plot actogram for the wheel running analysis between a C57B6/J male mouse (left) and an NPY::Venus Tg male mouse (right) (*n* = 3).

CMV::NPY::Venus plasmid DNA was provided by Takashi Tsuboi (University of Tokyo). It contained a CMV promoter, the human NPY open-reading frame (ORF) fused with Venus fluorescent protein, and the polyadenylation sites of simian virus (SV40) early genes in pcDNA3 (Figure 1A). A linearized DNA fragment of CMV::NPY::Venus, which was obtained via digestion with HindIII and XbaI, was microinjected into cryopreserved C57BL/6J mouse zygotes (CREA Japan Inc., Tokyo, Japan) to produce the Tg mice (RIKEN Kobe Campus, Hyogo, Japan) [26,27]. Linearized DNA fragments were microinjected into the cryopreserved C57BL/6J mouse zygotes to produce the Tg mice (Kobe RIKEN) [28].

### 2.2. Genomic PCR Screening of the Tg Mice

The CMV::NPY::Venus Tg mice were identified via genomic PCR using founder tail DNA with an Extract-N-Amp™ Plant PCR Kit (Merck Millipore, Burlington, MA, USA). The PCR primers 5′-ACCAGCGGAGGACATGG-3′ (the forward primer inside the NPY sequence) and 5′-GGCGGCGGTCACGAAC-3′ (the reverse primer inside the Venus sequence) produced approximately 650 bp fragments from the genomic DNA of the Tg mice using a GoTaq DNA polymerase (Promega, Madison, WI, USA). Electrophoresis was performed on a 1% agarose gel with a Midori Green Direct DNA stain (NIPPON Genetics Co., Ltd., Tokyo, Japan).

### 2.3. Animals

The CMV::NPY::Venus Tg hemizygous mice were maintained at 22 °C (in light/dark 12 h:12 h conditions with the lights on from 08:00 to 20:00). The mice were kept under specific pathogen-free conditions and tested according to the guidelines for the use of laboratory animals of the Toyohashi University of Technology (TUT) and ARRIVE. This study was performed in accordance with the recommendations for the Care and Use of Laboratory Animals stipulated by the Animal Research Committee of the TUT (protocol code DO2021-1). Both males and females were used, and no differences in the behaviors of the wheel-running activity rhythms were observed between the different constant dark and constant light conditions (*n* = 3).

The animals were divided into the following three groups: a group fed ad libitum, a group that fasted for 24 h, and a group that fasted for 60 h (male, *n* = 3). The animals were fed as a group ad libitum for 6 h and as a group ad libitum for 20 h after 60 h of fasting. Ad libitum food access was restored after fasting. The mice in the 60 h fasting group were assayed for their NPY levels in the SCN at 6 and 20 h after food access. We wanted to compare the secretion of NPY between 6 h (CT18) immediately after food recovery and 20 h (CT8) after recovery. The timing of the circadian rhythm of the NPY secretion should have been similar for the CT8 and CT18 groups under normal conditions. The NPY::Venus Tg and wild-type mice aged 4–7 months were weighed once per week. In Figure 1C, *n* = 1. In Figure 1D, *n* = 5. In Figure 1E, *n* = 1.

### 2.4. Wheel-Running Activity for the Mice

The mice were made to engage in a wheel-running activity using a wheel device in a cage (RWC-15, Melquest) under light–dark conditions (light/dark 12 h:12 h conditions, with the lights on from 08:00 to 20:00) for 2 weeks as a control after they had acclimatized for 1 week. DD (constant dark) conditions were induced for 2 weeks, followed by 1 week of light–dark conditions and 2 weeks of constant-light conditions. The wheel-running data were analyzed using ActMaster4M (Melquest, Toyama, Japan) to generate double-plot actograms. The results showed no differences between the males for the DD and LL conditions.

### 2.5. Preparation of the Mouse Brain Slices and Immunostaining

The mice were injected with 20% *w*/*v* urethane (0.4 mL/10 g of body weight) at ZT4. Next, 0.1 M of phosphate-buffered saline (PBS) containing 4% paraformaldehyde was circulated via the aorta. Coronal slices (40–60 μm thick) of the SCN were obtained using a vibratome slicer (Dosaka E.M., Kyoto, Japan). The brain slices were processed with blocking solution (PBS containing 0.1% Tween 20 with 1% bovine serum albumin and 22.52 mg/mL of glycine) for 60 min, immersed in 1 mM of rabbit anti-NPY primary antibody (Abcam, Cambridge, UK) for 60 min, and then immersed in 1 mM of goat anti-rabbit Y secondary antibody conjugated with Alexa Fluor 647 (Abcam) for 60 min.

### 2.6. Preparation of the Mouse Brain Slices and Transparency

The brain slices were prepared from the mice fed ad libitum, the group that fasted for 24 h, the group that fasted for 60 h, the group fed ad libitum for 6 h (after 60 h of fasting), and the group fed ad libitum 20 h (after 60 h of fasting), and we observed these using an A1 confocal microscope (Nikon, Tokyo, Japan). One brain slice was obtained from each mouse. Summed images of the z-stacks were created using NIS Elements software (Nikon Ver.5.11). NPY::Venus was excited with a 515 nm laser, and emissions were detected using a 520–540 nm bandpass filter. Alexa Fluor 647 was excited with a 647 nm laser, and emissions were detected using a long-pass filter. Images were acquired at 25× magnification (Lens, CFI Plan Apo λ, Nikon). The brains of the NPY::Venus Tg mice were immediately removed after euthanasia and placed in cooled PBS. Coronal slices (300–700 μm thick) of the SCN were prepared using a vibratome slicer. The brain slices were processed for transparency using SeeDB for the subsequent super-resolution microscopy analysis [28].

### 2.7. Fluorescence Observation by Confocal Microscopy

The each SCN slice of each NPY::Venus Tg line were observed using an A1 confocal microscope (Nikon, Tokyo, Japan) (males, *n* = 3). Summed images of the z-stacks were created using NIS Elements software (Nikon). NPY::Venus was excited with a 515 nm laser, and emissions were detected using a 520–540 nm bandpass filter. Alexa Fluor 647 was excited with a 647 nm laser, and emissions were detected using a long-pass filter. Images were acquired at 25× magnification (Lens, CFI Plan Apo λ, Nikon).

The number of peaks with fluorescence intensities of more than twice the average of the fluorescence intensities along a single line were quantified and subsequently summed across yellow 10 lines for a slice sample from one mouse. The number of NPY::Venus peaks over the threshold indicated the total number of cells in the 10 lines with fluorescence intensities more than twice the average of the fluorescence intensities

### 2.8. Fluorescence Observation by Super-Resolution Microscopy

The transparent brain slices were observed using a 60× immersion lens attached to an N-SIM structured illumination microscope (Nikon). Secretory granules containing NPY::Venus in the brain slices were imaged along the *Z*-axis. NPY::Venus was excited with a 515 nm laser and detected using a 520–540 nm filter. Images were acquired at 120× magnification (Lens, CFI Plan Apo λ, Nikon). The brain slices were also observed using a TCS SP8 STED (Leica, Leica Welt im Leitz-Park, Wetzlar, Germany).

### 2.9. Statistical Analysis

In all experiments other than the experiment that compared the body weights and lengths, the data were analyzed using the Tukey–Kramer method via R software (v.4.1.0.; R Foundation for Statistical Computing, Vienna, Austria). *p*-values of less than or equal to 0.05 were considered statistically significant. The error bars in the graphs show the means ± the standard deviations.

## 3. Results

### 3.1. Generation of the Tg Animals

The CMV::NPY::Venus plasmid contained the CMV promoter and the human NPY open-reading frame (ORF) fused with a Venus fluorescent protein, along with the polyadenylation sites of simian virus (SV40) early genes, and these were used to monitor NPY secretion using fluorescence signals (Figure 1A). The CMV::NPY::Venus Tg mice were identified through genomic PCR using tail DNA (Figure 1B). The exogenous NPY::Venus and NPY proteins stained with an anti-NPY antibody co-localized in most cells in the Tg SCN (Figure 1C). The NPY–Venus Tg mice were useful for investigating the spatiotemporal dynamics of the NPY secretory granules.

### 3.2. Selection of the NPY::Venus Tg Mice

**Figure 2:**
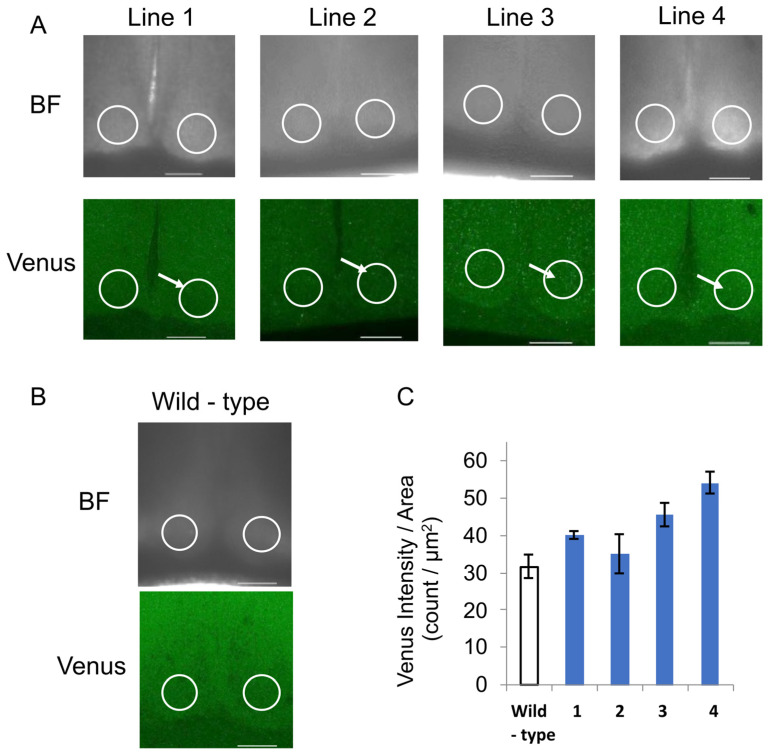
NPY::Venus expression levels in the SCN in lines 1–4 of the NPY::Venus Tg mice. (**A**) Images of the SCN slices of lines 1–4 of the NPY::Venus Tg mice acquired by confocal microscopy. BF: bright field. Venus: fluorescence field. Scale bar: 200 µm. The white circles show the center of the SCN where the nerve terminal projected into the SCN. The white arrows indicate cells with NPY::Venus secretory granules. (**B**) Images of an SCN slice of a wild-type mouse. BF: bright field. Venus: fluorescence field. (**C**) The average fluorescence intensity measured in the SCN area in integrated slice images from the Z-stacks (*n* = 3). In Figure 2A–C, *n* = 3.

We generated NPY::Venus Tg mice in which a of fusion the protein of human NPY tagged with Venus, a mutated yellow fluorescent protein, was ubiquitously overexpressed under the control of the CMV promoter (Figure 2A). Four lines of NPY::Venus Tg mice (lines 1–4) were obtained via the transgene PCR screening of mouse tail DNA (Figure 2A,B). Lines 2 and 4 had the lowest and highest expression of NPY::Venus, respectively, according to the Venus signals in the center of the SCN where the NPY was secreted. Line 1 was used for subsequent experiments because Lines 3 and 4 were prone to overexpressing NPY::Venus while Line 2 expressed NPY::Venus unreliably, with large individual error values (Figure 2C). We confirmed that the development and activity rhythms of the Line 1 mice were similar to those of wild-type mice (Figure 1D,E).

### 3.3. Imaging of the SCN Slices of the NPY::Venus Tg Mice

**Figure 3:**
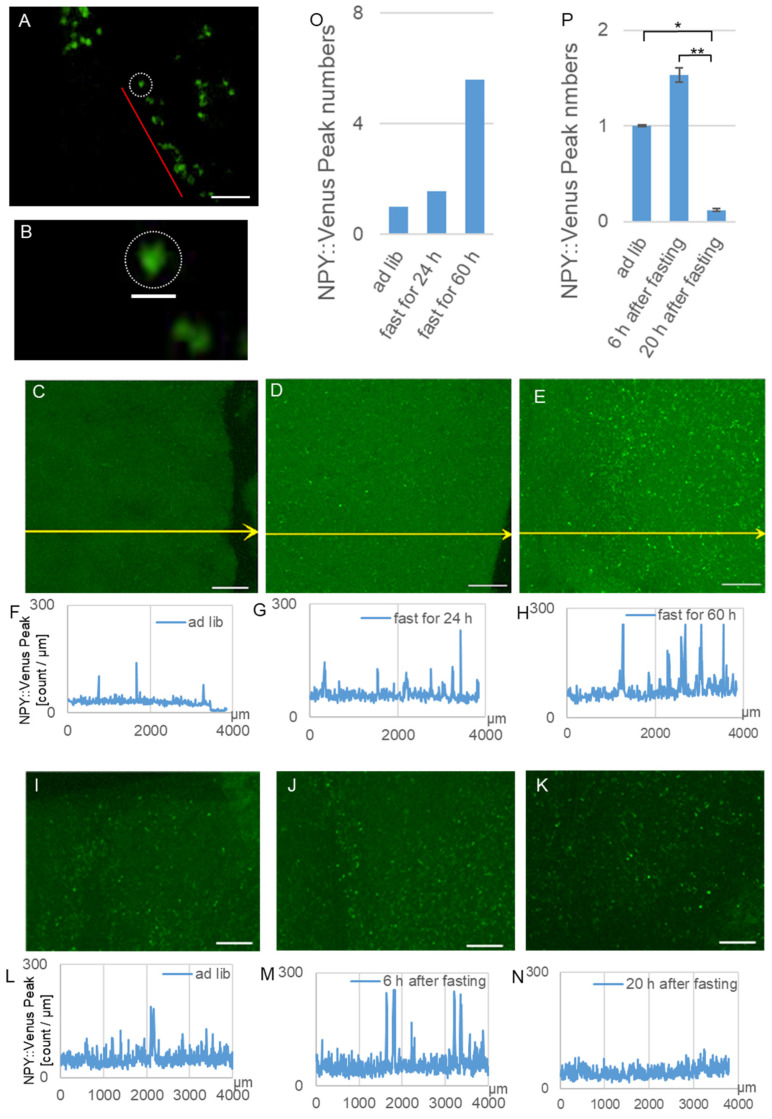
Fluorescence imaging of the NPY::Venus-containing secretory granules by super-resolution microscopy in the fasted mice. (**A**) Fluorescence image of the perinuclear NPY::Venus-containing secretory granules in an SCN slice acquired by super-resolution microscopy. Red line: secretory granules arranged in series. Scale bar: 1 µm. (**B**) Magnified image of the secretory granules along the red line around the SCN nucleus denoted by a white circle. Scale bar: 100 nm. (**C**) Image of the NPY::Venus fluorescence in the SCN of an NPY::Venus Tg mouse fed *ad libitum*. Scale bar: 500 µm. (**D**) Image of the NPY::Venus fluorescence in the SCN of an NPY::Venus Tg mouse that fasted for 24 h. (**E**) Image of the NPY::Venus fluorescence in the SCN of an NPY::Venus Tg mouse that fasted for 60 h. (**F**) Graph of the fluorescence intensity along the yellow arrow in (**C**). (**G**) Graph of the fluorescence intensity along the yellow arrow in (**D**). (**H**) Graph of the fluorescence intensity along the yellow arrow in (**E**). (**I**) Image of the NPY::Venus fluorescence in the SCN of a mouse fed *ad libitum*. Images of the NPY::Venus fluorescence in the SCN of mice fed (**J**) 6 h after fasting for 60 h and (**K**) 20 h after fasting for 60 h. (**O**) Total numbers of the cells in 10 yellow lines (shown in (**C**–**E**)) whose fluorescence intensities reflect the following three groups: the fed *ad libitum* group and the groups that fasted for 24 and 60 h (shown in (**F**–**H**)). (**P**) Total numbers of cells in 10 lines whose fluorescence intensities reflect the following three groups: the fed *ad libitum* group and the groups fed at 6 and 20 h after fasting for 60 h (shown in (**L**–**N**)). ** *p* < 0.005, * *p* < 0.01, Tukey–Kramer Test. In Figure 3A,B, *n* = 1. In Figure 3C–H,O, *n* = 1. In Figure 3I–N,P, *n* = 3.

In the cranial nerve cells of the NPY::Venus Tg mice, super-resolution microscopy revealed that many secretory granules containing NPY::Venus had congregated in a line around the SCN (Figure 3A,B). The NPY::Venus protein may be condensed in secretory granules in neurons and localized to the presynaptic membrane in a line. The diameters of the fluorescent particles were similar to those of the secretory granules and were reported to be approximately 100 nm. We observed that secretory granules with NPY::Venus were delivered to the SCN of the Tg mice.

### 3.4. Comparison of the NPY::Venus Expression Between the Feeding Condition Groups

Cells containing secretory granules were investigated in the groups that were fed ad libitum, fasted for 24 h, and fasted for 60 h. Cells in which secretory granules were strongly labeled with NPY::Venus in the SCN slices of the NPY::Venus Tg mice were observed using confocal microscopy (Figure 3C–H). More secretory granules containing NPY::Venus were observed in the SCN in the groups that fasted for 24 h and 60 h than in the group fed ad libitum, as stronger fluorescence signals of NPY::Venus were detected. The number of secretory granules containing NPY::Venus increased approximately 1.3-fold in the group that fasted for 24 h and 3.6-fold in the group that fasted for 60 h, respectively (Figure 3O).

When food was provided again after the mice had been subjected to 60 h of fasting, the quantity of NPY around the SCN was counted in the same way as shown in Figure 3F–H at 6 and 20 h after fasting, and it was compared with the group fed ad libitum without fasting (Figure 3I–N). When food was presented for 6 h after fasting, the number of secretory granules decreased 1.5 times. Moreover, it subsequently decreased to this level, corresponding to 0.3 times that for the control, when food was re-presented for 20 h after fasting (Figure 3P).

## 4. Discussion

Under typical free-feeding conditions, the SCN functions as a central clock, entraining body rhythms to light–dark cycles and orchestrating the circadian rhythms of all peripheral tissues and behaviors. However, under 4 h of RF under light conditions, the FEO entrains the rhythms of peripheral digestive tissues and behaviors to an organism’s feeding schedule and becomes independent of SCN control [4]. Then, the SCN continues to maintain the phases of circadian rhythms based on the light–dark cycle, and its phases are not influenced by feeding timing [5,8,28,29,30,31,32]. In other words, during RF, the FEO emerges and severs the body’s rhythms from the SCN’s circadian control. Upon returning to ad libitum feeding, the SCN rhythm and body rhythm gradually synchronize.

NPY is a neurotransmitter released from the IGL into the SCN that transmits feeding-related signals [11,12,13,14,15,16,17,18,19] and inhibits the pathway through which the SCN responds to light [18,19]. We hypothesized that NPY could provoke the FEO to be uncoupled from the SCN and regulate rhythms, including behavioral patterns during fasting.

In this study, we developed a novel Tg mouse model in which the NPY::Venus fusion protein accumulated in secretory granules, allowing the spatiotemporal visualization and quantitative estimation of NPY secretion based on the number of granules exhibiting Venus fluorescence [20,21,22,33]. However, it is possible that the ubiquitous expression of the exogeneous NPY::Venus gene by the CMV promoter had an effect on the transcription of endogenous NPY expression in cells. If transcriptional modification plays a role in the feeding-dependent regulation of NPY levels, these transgenic animals may not accurately reflect the endogenous regulation of NPY. To minimize the impact of the exogenous NPY::Venus gene on that of native NPY, we selected a transgenic line where the expression of NPY::Venus was comparatively modest and the co-localization of NPY and NPY::Venus was subsequently confirmed via immunostaining.

Line 1 was used in this study because these mice moderately expressed NPY::Venus and exhibited normal behavioral rhythms (Figure 1D,E). NPY::Venus signals in the secretory granules gradually accumulated in the SCN slices of Line 1 until 60 h of fasting had elapsed. Then, the quantity of secretory granules containing NPY::Venus diminished to approximately one-third of that in the group fed ad libitum for 14 h (6–20 h) after food re-presentation (Figure 3P), suggesting that these secretory granules could have been partly released from pre-synapses and then disappeared.

**Figure 4:**
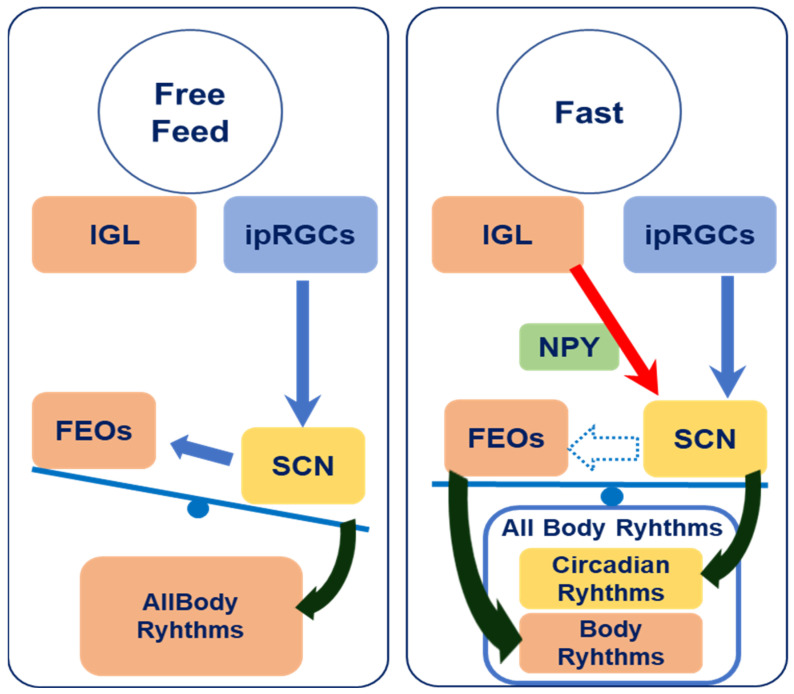
Schematic representation of the relationship between the FEO and SCN. In the free-feeding condition, body rhythms are controlled by the SCN. In contrast, in a fasting condition, IGL neurons induce body rhythms to work independently of the SCN by NPY and entrain them to feeding timing. All arrows indicate to effect on tissues and rhythms.

The conceptual framework of the interaction between the SCN and FEO under ad libitum and fasting conditions is illustrated in Figure 4. This interaction could be affected by elevated NPY secretion around the SCN. The FEO is regulated by the SCN under free-feeding conditions (left side), while feeding-related external cues attenuate the input of photo signals to the SCN and/or diminish the dominance of the SCN over the FEO when feeding requirements are increased (right side) [34]. The FEO may regulate body rhythms independently of the SCN. In a fasting state, the FEO and SCN could become desynchronized, resulting in the desynchronization of the peripheral tissues and the central SCN, as shown on the right side of Figure 4.

In the future, in vivo imaging of NPY::Venus Tg mice can elucidate the intricate relationship between the FEO and SCN through the real-time spatiotemporal observation of NPY secretion in the brain when feeding requirements are changed. We have yet to dynamically observe whether the NPY secretory granules in the presynaptic membrane projecting to the SCN are, indeed, secreted into the SCN. However, we can monitor the disappearance of secretory granules within the SCN slices through TIRF fluorescence imaging [20]. This methodology will enable us to track the quantity of NPY secretory granules over time and in real time. Future directions may involve the application of real-time fluorescence imaging in both SCN and IGL brain slices using TIRF fluorescence microscopy, as well as in vivo imaging via fiber microscopy under various experimental conditions. The quantity of NPY-secreting granules within the nuclei of IGL neurons is correlated with those located near the SCN.

From the perspective of chronopharmacology, the rhythmic phases of the target tissues are vital for determining the efficacy of a drug. Consequently, it is of significant medical importance to comprehend the mechanisms whereby the timing of food intake or light–dark conditions influence the rhythmic phases of each target tissue.

## Data Availability

Data are contained within the article.

## Abbreviations

The following abbreviations are used in this manuscript:

SCN	the suprachiasmatic nucleus
FEO	the food-entrainable circadian oscillator
NPY	neuropeptide Y
IGL	intergeniculate leaflet
Tg	transgenic
*Per*	*Periods*
*Per1*::*luc*	*Per1 promoter*::*luciferase*
RF	restricted feeding
FF	free feeding
ZT	Zeitgeber time
FAA	food anticipatory activity
ORF	open reading frame
SV	simian virus
TUT	Toyohashi University of Technology
DD	constant dark
PBS	phosphate-buffered saline
